# Osteoporosis among Postmenopausal Women Attending the Orthopedics Department of a Tertiary Care Hospital: A Descriptive Cross-sectional Study

**DOI:** 10.31729/jnma.6031

**Published:** 2021-05-31

**Authors:** Sunil Panta, Madhu Neupane, Shrawan Kumar Thapa, Kalyan Sapkota

**Affiliations:** 1 Department of Orthopedics, Bharatpur Hospital, Chitwan, Nepal; 2 Department of Radiodiagnosis, Imaging and Nuclear Medicine, BP Koirala Memorial Cancer Hospital, Chitwan, Nepal; 3 Department of Internal Medicine, Bharatpur Hospital, Chitwan, Nepal

**Keywords:** *bone mineral density*, *osteoporosis*, *postmenopausal*

## Abstract

**Introduction:**

Osteoporosis and resulting fracture is a major public health concern worldwide. With increase in life expectancy, osteoporosis and fragility fracture is expected to be more prevalent. It is associated with high patient morbidity, while hip and vertebral fractures have high mortality. The real burden of the problem is yet to be established in developing countries like Nepal. This study aims to find out the prevalence of osteoporosis among postmenopausal women visiting a tertiary care hospital.

**Methods:**

This descriptive cross-sectional study was conducted among 89 postmenopausal women attending at Orthopedic Outpatient Department of Bharatpur Hospital from 1st January 2019 to 30th December 2019. The ethical clearance was taken from the Institutional Review Committee of Bharatpur Hospital. Convenience sampling technique was used. Bone Mineral Density was estimated with dual energy x-ray absorptiometry scan. Statistical Package for Social Science was used for analysis. Point estimate at 95% Confidence Interval was calculated along with frequency and proportion for binary data.

**Results:**

Out of 89 postmenopausal women, 29 (32.58%) (Confidence Interval = 32.48-32.68) women had osteoporosis. The mean age and Bone Mineral Density were 62.16±8.17 years and 0.968±0.14 g/cm^2^ respectively. The women with history of fragility fracture had low bone mineral density.

**Conclusions:**

Prevalence of osteoporosis was high among postmenopausal women. Women with history of fragility fracture are at increased risk of another fragility fracture. It is hence necessary to have awareness programs and early screening to minimize the magnitude of morbidity and mortality associated with osteoporosis.

## INTRODUCTION

Osteoporosis is a systemic skeletal disorder that is characterized by low bone mass and micro architectural deterioration of bone tissue, with a consequent increase in bone fragility and susceptibility to fracture. Incidence of osteoporosis and resulting fracture increases with advancing age and are associated with high morbidity and mortality.^1-4^ Postmenopausal, post-hysterectomy status women and some other factors are responsible for low bone mineral density (BMD).^5^ Among different methods of measurement of BMD, Dual energy x-ray absorptiometry (DEXA) scan is regarded as gold standard.^6,7^

There is worldwide variation of data on incidence and prevalence of osteoporosis.^1,8-10^ Data from developing countries are scarce. Very few studies have been done in our part of the world using gold standard DEXA technology.^11,12^ Prevalence of osteoporosis is therefore essential to formulate strategies to help and reduce patient morbidity and mortality.

This study aims to find out the prevalence of osteoporosis among postmenopausal women.

## METHODS

This is a descriptive cross sectional study conducted at the Orthopedic Department of Bharatpur Hospital over a period of 1 year, from 1st January 2019 to 30th December 2019. The ethical clearance was taken from the Institutional Review Committee (IRC) of Bharatpur Hospital. All postmenopausal women excluding women with skeletal deformity, under hormone replacement therapy and corticosteroid therapy, with co-morbidities, smokers and post hysterectomy, were counseled for Bone Mineral Density measurement with DEXA scan. Sample size was determined by using the convenience sampling technique among the patients meeting inclusion criteria.

Sample size was calculated using formula,


$$\begin{array}{ll} \text{n} & \text{= }{\text{Z}}^{\text{2}}\times \text{p}\times \text{q}/{\text{e}}^{\text{2}}\\ & \text{= }{\left(1.96\right)}^{\text{2}}\times 0.50\times (1-0.50)/{(\text{0.11)}}^{\text{2}}\\ & \text{= }79 \end{array}$$

Where,

n = minimum required sample size,
Z = 1.96 at 95% Confidence Interval
p = prevalence taken as 50% for maximum sample size
q = 1-p
e = margin of error, 11%

Adding 10% non-response rate, sample size= 87. However 89 women were included in the study. Informed written consent was taken. All historical information was obtained using a structured questionnaire. Variables like age, age at menopause and duration of menopause were recorded.

Previous history of fracture after the onset of menopause was recorded. Only vertebral, hip and distal radius fractures were included as fragility fracture. Height and weight was taken and recorded. DEXA scan was done using OsteoSys_Primus machine at Osteolife Thyroid Healthcare Pvt. Ltd. BMD and T score were calculated. Data interpretation was done as per WHO guideline. Normal: T score ≥-1, Osteopenia: T score -1 to -2.5, Osteoporosis: T score ≤-2.5 and below.^13^ BMI was calculated as weight in kilograms/ height in meter square.

Data collected from structured questionnaire and DEXA scan report were compiled and analyzed using IBM Statistical Package for the Social Sciences 20.0 version software.

## RESULTS

Out of 89 postmenopausal women, 29 (32.5%) (Confidence Interval = 27.70-27.89) women had osteoporosis. Mean age was 62.16±8.17 years. Mean of different demographic variables were calculated. Mean age at menopause and duration of menopause were 48.10±3.68 and 14.06±9.13 in years respectively. Mean BMI was 24.88±5.10, mean whole body BMD was 0.96±0.14g/cm^2^ and mean T score was -1.49±1.62 (Table 1).

**Table 1:** Demographic and clinical characteristics (n = 89).

Variables	Mean±SD	Minimum	Maximum
Age in years	62.16±8.17	45	85
Age at menopause in years (n = 89)	48.10±3.68	35	55
Duration of menopause in years (n = 89)	14.06±9.13	1	40
BMI	24.88±5.10	15.01	42.98
Whole body BMD	0.96±0.14	0.67	1.30
T score	-1.49±1.62	-5	2.6

Out of 89, 29 (32.58%) were osteoporotic, 31 (34.83%) were osteopenic and rest were found to have normal bone density (Table 2).

**Table 2:** Prevalence of osteoporosis (n = 89).

Osteoporosis	n (%)
Normal	29 (32.58)
Osteopenia	31 (34.83)
Osteoporosis	29 (32.58)

Mean BMD was found to be 0.844±0.15, 0.903±0.17 and 0. 876±0.16 of lumbar spine, right hip and left hip respectively (Table 3).

**Table 3:** Regional BMD variation (n = 89).

BMD (gm/cm^2^)	Mean±SD	Maximum	Minimum
Lumbar spine BMD	0.844±0.15	1.215	0.443
Right hip BMD	0.903±0.17	1.274	0.471
Left hip BMD	0.876±0.16	1.177	0.510

**Figure 1.**
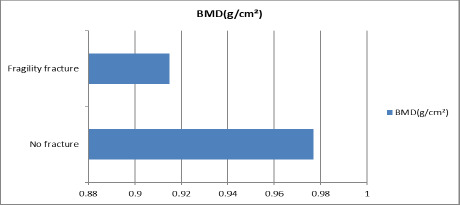
Previous Fragility Fracture and BMD.

Among 89 women, 13 (14.61%) had a history of fragility fracture after the onset of menopause. Women with previous history of fracture had low bone mineral density (mean 0.915±0.15) compared to non-fracture group (mean 0.977±0.13) (Figure 1).

## DISCUSSION

The present study was conducted to find out the prevalence of osteoporosis among postmenopausal women. Most of the studies on Osteoporosis prevalence have been done with quantitative ultrasound technology. BMD measurement with gold standard DEXA scan is very rare in our part of the world.

Among 89 women included in this study, 29 (32.58%) had normal bone density while rest 60 women were found to have low BMD with 29 (32.58%) being Osteoporotic and 31 (34.83%) being Osteopenic. Prevalence of osteoporosis at Chitwan was 26.2% in the study done by Dhakal KS, Dhakal S, Aryal B, 2012.^12^ In another study by Chaudhary NK et al, 2019^11^ prevalence of Osteoporosis in Kathmandu was found to be relatively higher (37.3%).

In most of the studies there is a significant trend of decreasing bone density with an increase in age and duration of menopause which are similar to findings in our study. Women with low BMI had low BMD in our study. In studies done by Salamat MR, Salamat AH, Abedi I, Janghorbani^10^ and Woolf AD, Pfleger B,^14^ also found similar findings. Our finding is in variance with Sahu S, Mohapatra I, Sharma P,^15^ and Munshi R, Kochhar A, Garg V,^16^ where they found decrease in BMD with increase in BMI.

Our study showed low BMD at the lumbosacral spine than hip. Mean Lumbosacral spine BMD is 0.844±0.15, right hip BMD is 0.903±0.17 and left hip BMD is 0.876±0.16. This finding is supported by another study done by Mounach A, et al. 2009^17^ and Kadam NS, Chiplonkar SA, Khadilkar AV, Khadilkar VV, 2018.^9^ This regional disconcordance in BMD may be attributable to the fact that spine contains more trabecular bone. Another explanation could be that weight-bearing causes rise in bone density especially in the femur and hip region.^8,9^

Women with previous history of fragility fracture were found to have low BMD than those with no history fragility fracture. Mean BMD of fragility fracture group is 0.915±0.15 and those of non-fracture group is 0.977±0.13. This indicates that women with fragility fracture are at added risks of another fragility fracture. In a study done by Akesson K. et al, 201318 also found women presenting with fragility fracture are two times as likely to suffer another fracture as their peers.

Detailed data on other risk factors like co-morbidities, smoking status, nutritional status was not included in the study, which could influence the findings. More number of postmenopausal women has to be included in the study for more precise result. This is a study done in patient visiting to hospital only, general prevalence could be different. Large scale, multi-centric, randomized sampling study is needed to minimize the bias and establish the real burden of the problem.

## CONCLUSIONS

Postmenopausal women are at high risk of osteoporosis and osteopenia. Women with history of fragility fracture are at increased risk of another fragility fracture. Early detection of osteoporosis using DEXA can be a good screening tool. There is need of awareness programs to these risk groups and minimize the possible fracture risk.
